# Supplementary material for: 2D facial landmark localization method for multi-view face synthesis image using a two-pathway generative adversarial network approach
Source: PeerJ Comput Sci. 2022 Feb 16;8:e897. doi: 10.7717/peerj-cs.897 (PMC9044338; doi:10.7717/peerj-cs.897)
Supplement: Supplemental Information 1 — The layers of the GAN model. [file peerj-cs-08-897-s001.pdf]

Appendix:

Table 1:

Patches size or shape guideline. W and H is the image width and height dimension.

Method	Dataset	Profile image size	Patches shape size
TP-GAN	• Multi-PLE<br>• FEI	$128 \times 128 \times 3$<br>$128 \times 128 \times 3$	$Eyes\ shape: W40 \times H40$<br>$Nose\ shape: W32 \times H40$<br>$Mouth\ shape: W32 \times H48$
LFMTP-GAN	• Multi-PLE<br>• FEI	$128 \times 128 \times 3$<br>$128 \times 128 \times 3$	

Table 2:

The $\{G_{\theta_E^l}, G_{\theta_D^l}\}$ structure of the local pathway. W and H is the image width and height dimension.

Layer	Input	Filter size	Output size
		$Local\ encoder\ G_{\theta_E^l}$	
conv0	$---$	$3 \times 3 / 1$	$w \times h \times 64$
conv1	$---$	$3 \times 3 / 2$	$w/2 \times h/2 \times 128$
conv2	$---$	$3 \times 3 / 2$	$w/4 \times h/4 \times 256$
conv3	$---$	$3 \times 3 / 2$	$w/8 \times h/8 \times 512$
		$Local\ decoder\ G_{\theta_D^l}$	
deconv0	$---$	$3 \times 3 / 2$	$w/4 \times h/4 \times 256$
conv4	conv2	$3 \times 3 / 1$	$w/4 \times h/4 \times 256$
deconv1	$---$	$3 \times 3 / 2$	$w/2 \times h/2 \times 128$
conv5	conv1	$3 \times 3 / 1$	$w/2 \times h/2 \times 128$
deconv2	$---$	$3 \times 3 / 2$	$w \times h \times 64$
conv6	conv0	$3 \times 3 / 1$	$w \times h \times 64$
feat	conv6	$3 \times 3 \times 1$	$w \times h \times 3$

Table 3:
The $\{G_{\theta_E^g}\}$ structure of the global pathway.

Layer	Filter size	Output size
conv0	$7\times 7 / 1$	$128\times 128\times 64$
conv1	$5\times 5 / 2$	$64\times 64\times 64$
conv2	$3\times 3 / 2$	$32\times 32\times 128$
conv3	$3\times 3 / 2$	$16\times 16\times 256$
conv4	$3\times 3 / 2$	$8\times 8\times 512$
fc1	$---$	512
fc2	$---$	256

Table 4:
The $\{G_{\theta_D^g}\}$ structure of the global pathway. The *convs* in *input* column refer to those in *Table* .
I^P is the profile image.

Layer	Input	Filter size	Output size
feat8	fc2.x	$---$	$8\times 8\times 64$
feat32	$---$	$3\times 3 / 4$	$32\times 32\times 32$
feat64	$---$	$3\times 3 / 2$	$64\times 64\times 16$
feat128	$---$	$3\times 3 / 2$	$128\times 128\times 8$
deconv0	feat8, conv4	$3\times 3 / 2$	$16\times 16\times 512$
deconv1	conv3	$3\times 3 / 2$	$32\times 32\times 256$
deconv2	feat32, conv2, I^P	$3\times 3 / 2$	$64\times 64\times 128$
deconv3	feat64, conv1, I^P	$3\times 3 / 2$	$128\times 128\times 64$

Table 5:
The structure of 5, 6 and 7 ConvNet-layers. I^P is the profile image.

Layer	Input	Filter size	Output size
conv5	feat128, conv1, local, I^P	$5 \times 5 / 1$	$128 \times 128 \times 64$
conv6	$---$	$3 \times 3 / 1$	$128 \times 128 \times 32$
conv7	$---$	$3 \times 3 / 1$	$128 \times 128 \times 3$
